# Antibiotic-Mixed Cement Filling for Chronic Osteomyelitis

**DOI:** 10.3390/jpm15050187

**Published:** 2025-05-06

**Authors:** Seung-Hwan Park, Young Rak Choi, Inyong Jeong, Ho Seong Lee

**Affiliations:** 1 Department of Orthopedic Surgery, Chung-Ang University Hospital, College of Medicine, Chung-Ang University, 102, Heukseok-ro, Dongjak-gu, Seoul 06973, Republic of Korea; sh-first@hanmail.net (S.-H.P.); jeonginyong@cauhs.or.kr (I.J.); 2 Department of Orthopedic Surgery, Asan Medical Center, University of Ulsan College of Medicine, 88, Olympic-ro 43-gil, Songpa-gu, Seoul 05505, Republic of Korea; jeanguy@hanmail.net

**Keywords:** osteomyelitis, debridement, cementoplasty

## Abstract

**Background/Objectives:** Traditional treatment for chronic osteomyelitis is temporary implantation of antibiotic-impregnated cement beads, followed by bone grafting after the infection is controlled. In this way, a staged operation is needed, and undergoing repetitive general anesthesia is a burden. Moreover, damage to the soft tissue at the surgical site due to several incisions is a concern. This study was conducted to investigate the outcomes of one-stage antibiotic-mixed cement blocks, instead of beads, used as a primary salvage procedure to treat chronic osteomyelitis of the foot, ankle, and lower leg. **Methods:** Twenty patients with chronic osteomyelitis of the leg and foot were included. They underwent complete debridement of the infected bone, and antibiotic-mixed cement fillings were placed into the defected bone space. Full-weight-bearing activities were allowed immediately after surgery. **Results:** For 16 of the 18 patients, infection was controlled after one-time surgery. Repeat antibiotic cement-filling surgery was necessary for two patients. Two-staged surgery with continuous irrigation and cement filling was necessary for one large tibial lesion. Conversion into arthrodesis of the metatarsophalangeal joint was necessary for metatarsal head infection. **Conclusions**: One-stage surgery with complete debridement and antibiotic-mixed cement filling is a simple and effective procedure for treating intractable chronic osteomyelitis, which makes full-weight-bearing walking possible immediately after surgery.

## 1. Introduction

Surgical debridement, dead space filling with antibiotic-impregnated synthetic materials, and subsequent systemic antibiotic therapy are the traditional treatments for chronic osteomyelitis. Antibiotic-impregnated cement beads are considered the standard vehicle for local antibiotic delivery. Two-step surgery is performed to treat most cases of chronic osteomyelitis. Following the control of infection through debridement of infected tissue and temporary filling of the dead space with antibiotic cement beads, the second stage of cement removal and bone grafting is performed [1,2,3,4,5,6,7]. After its role of local antibiotic delivery is met, the cement remains as a foreign body and would negatively affect the treatment of chronic osteomyelitis; thus, the cement is removed, and bone grafting is performed.

Antibiotic-impregnated cement has been used as an option for local antibiotic diffusion for treating musculoskeletal infections. In a sentinel paper, Buchholzand Engelbrecht reported that penicillin, erythromycin, and gentamicin mixed into the cement used to affix prostheses to the bone provide high concentrations of antibiotics for extended periods of time, facilitating the use of antibiotics in infection prophylaxis for joint arthroplasty [8]. In addition to this role, local antibiotic therapy has been instituted for treating arthroplasty infections, prophylaxis open fractures, and treating chronic osteomyelitis. In 1979, Klemm created gentamicin-impregnated beads and used them to occupy dead space after debridement of an infected bone. In more than 100 patients, a cure rate of 91.4% was achieved [9]. In most cases of antibiotic-impregnated bone cement treatment, the cement is inserted in a bead form. After the infection is controlled, cement beads are removed, and additional bone grafting is performed.

In the last few years, the most common use of cement block followed by grafting to treat chronic osteomyelitis or bone defects has been the Masquelet technique [10]. In another way, a treatment that uses biodegradable antibiotic carriers such as Stimulan and Cerament V/G to control infection and induce bone formation in the defected area is also being implemented [11,12,13]. However, there are some disadvantages to these treatments. Antibiotic bead insertion and the Masquelet technique require several-stage surgery [10]. The biodegradable antibiotic carriers have a weak initial strength, so additional fixation is often needed. There is also a report that antibiotic carriers, the main component of calcium sulphate, can cause hypercalcemia [13]. It is not easy to fill large bone defects due to the characteristics of the formulation. There is also a problem of high cost. If osteogenesis is not performed properly in both methods, even if the infection is controlled, instability of the bone defect may remain.

We considered a one-stage surgery by molding and filling the dead space after debridement rather than temporarily inserting an antibiotic cement in the form of a bead. Few studies have been conducted on what would happen if cement is left behind. Unlike cement beads, cement blocks fill the defected bone site, making full-weight-bearing activities immediately after surgery.

Some studies have reported that antibiotic cement can play a significant role not only as a temporary vehicle for local antibiotic delivery but also as a spacer in ankle joint replacement surgery [14,15]. Recent advances in chronic osteomyelitis treatment have highlighted the effectiveness of antibiotic-laden cement in both infection control and structural support. For example, Canavese et al. (2017) demonstrated high success rates in pediatric patients using antibiotic-laden cement spacers combined with bone graft substitutes, emphasizing the potential of single-stage procedures in managing this condition [16].

This study was conducted to investigate the outcomes of debridement and dead space filling with antibiotic-impregnated cement block used as the primary salvage procedure to treat chronic osteomyelitis of the foot, ankle, and lower leg and to determine the problems that may occur when the cement block is left and the results related to full-weight-bearing walking.

## 2. Materials and Methods

### 2.1. Study Design and Participants

This retrospective study was approved by our Institutional Review Board (approval number: 2020-0031). Patients with chronic osteomyelitis of the tibia or bones of the foot and ankle, who underwent debridement of the infected bone and filling with an antibiotic-impregnated cement block in the defected bone space, were included in this study. Eighteen patients were treated by two surgeons from September 2004 to November 2017. All patients included in this study were treated using a standardized surgical protocol with the same type of antibiotic-loaded bone cement and mixing method. The procedures were performed by two orthopedic surgeons following the same protocol. They were diagnosed with chronic osteomyelitis. The diagnosis was made according to physical findings of redness, swelling, and repetitive discharge, radiographs, and laboratory results. In addition, three sterile site specimens were collected in the operating room and then a culture identification test was performed [17]. Patients with diabetic foot or immune-compromised patients were excluded from this study.

Of the 18 patients, 6 were female and 12 were male. The mean follow-up period was 93 months (range, 40–210 months) after antibiotic-impregnated cement filling. The mean age at surgery was 52.6 years (range, 23–79 years). The causes of infection were as follows: in three patients, infection occurred after open fractures, and in one patient, infection occurred after soft tissue laceration. Ten patients had postoperative infections: among them, seven occurred after open reduction and internal fixation of closed fractures, one occurred after supramalleolar osteotomy, one occurred after excision of skin tumor of the proximal tibia, and one occurred after metatarsal osteotomy for hallux valgus. In two patients, foot infection was caused by a puncture wound. Two patients had no history of trauma or surgery (Table 1).

The mean period between the initial diagnosis of infection and antibiotic-impregnated cement-filling surgery was 38.3 months (range, 2–240 months). All patients were initially diagnosed at other hospitals before visiting our clinic. Twelve patients underwent surgery, including incision and drainage (I&D) and anti-bead insertion. The other six patients received antibiotic treatment after I&D only. The average number of operations for infection control was 1.8 (range, 1–10) before antibiotic-impregnated cement-filling surgery in our hospital.

### 2.2. Operative Technique

After the incision over the draining sinus or infected skin, the implant was removed. All devitalized and infected tissues were debrided, and exposed osseous lesions were completely curetted. The osseous surfaces were debrided using a curette until healthy osseous structures were visible. Three tissue samples for culture and biopsy were obtained. Antibiotic-loaded bone cement containing gentamicin was mixed with 1 g of vancomycin and used for each patient. One vial (1 g) of vancomycin was mixed with 40 g of cement per case. The gentamicin content in the cement is approximately 1.25% by weight. The total antibiotic content was kept below 5% to avoid compromising the mechanical strength of the cement, in line with the commonly accepted threshold of 10%. Covering a wide spectrum of Gram-negative and Gram-positive bacteria, including methicillin-resistant *Staphylococcus aureus*, was intended. The cement powder was evenly mixed with vancomycin powder and solvent and was inserted into the dead space when it was in a dough state. A suction drain was inserted, followed by wound closure.

### 2.3. Postoperative Care

No immobilization was deployed in all patients. Moreover, full-weight-bearing walking as tolerated was allowed immediately after surgery. The result of the tissue culture was confirmed, and antibiotics were used accordingly. Intravenous antibiotics were administered during hospitalization, and oral antibiotics were additionally administered as needed after patient discharge.

### 2.4. Evaluation

#### 2.4.1. Clinical Evaluation

Clinical history was based on questionnaires, completed by the patients, which asked about the duration of the infection and number of surgeries before antibiotic-impregnated cement filling. Postoperative physical findings, clinical symptoms, and duration of antibiotic therapy were evaluated.

#### 2.4.2. Radiographic Evaluation

A simple radiograph was obtained preoperatively, and if necessary, a three-phase bone scan or enhanced magnetic resonance imaging (MRI) and enhanced computed tomography (CT) were additionally performed. Postoperatively, simple radiographs were used to check the lesion site and assess cement position, cement breakage, bone loss, and osteolysis around the inserted cement.

#### 2.4.3. Laboratory Evaluation

The erythrocyte sedimentation rate (ESR) and C-reactive protein (CRP) levels were checked preoperatively and postoperatively until the values were normalized. Bacterial culture was performed by taking samples of infected tissue during surgery. The samples obtained by swapping the skin were not used because they were unreliable due to contamination.

## 3. Results

### 3.1. Clinical Outcomes

In this study, the involved bones included the proximal tibia (n = 1), middle tibia (n = 1), distal tibia (n = 5), calcaneus (n = 7), cuboid (n = 1), medial cuneiform (n = 1), and first metatarsal bone (n = 2). (Table 1) According to the anatomical classification of the Cierny–Mader grade system used for diagnosis of chronic osteomyelitis, all these patients were stage III [18,19].

On average, the suction drain was removed 8.4 days (range, 3–21 days) postoperatively. In 16 of the 18 patients (88.9%), infection was controlled after one-time surgery by complete debridement and antibiotic-impregnated cement filling. Repeat antibiotic cement-filling surgery was necessary in two patients (cases 2 and 11). In a patient with chronic osteomyelitis of the calcaneus, pinpoint serous discharge persisted for 4 weeks; after antibiotic-impregnated cement-filling surgery, infection improved based on laboratory and physical examination findings. After repeat surgery with marginal debridement of the surgical wound, pinpoint discharge was discontinued (case 11). In a patient with an infection of the first metatarsal head, we converted arthrodesis of the metatarsophalangeal joint because of persistent serous discharge (case 17). In a patient with a cuboid infection involving the tarsometatarsal joint, serous discharge persisted for 30 days and healed eventually without additional surgery (case 15). In one case of tibia shaft infection, two-step surgery was performed because the lesion size was too large. First, we irrigated the wound continuously with saline at the bedside for 7 days after the removal of the previously inserted cement bead and then filled the tibia with an antibiotic-mixed cement block. The patient has been walking well even with a bowing leg for 16 years without recurrence of infection (case 6).

### 3.2. Radiographic Outcomes

Simple radiographs were taken preoperatively in all patients. Bone scan was performed in five patients, enhanced MRI in 11 patients, and CT in six patients. Two patients had all three tests. The osteolytic lesion was visible on the preoperative simple radiograph in nine patients, and no clear lesion was observed in the other 11 patients. The lesion of chronic osteomyelitis was observed on the additional enhanced MRI or CT.

### 3.3. Laboratory Outcomes

The timing of the normalization of the ESR and CRP levels is listed in Table 1. The causative organisms were identified in 15 of the 18 patients. Three infections were due to Methicillin-sensitive *Staphylococcus aureus*, four were caused by Methicillin-resistant *Staphylococcus aureus*, two were due to Pseudomonas aeruginosa, one was caused by Escherichia vulneris, one was caused by Streptococcus agalactiae, one was due to Salmonella typhi, one was due to Achromobacter xylosoxidans, one was caused by Mycobacterium tuberculosis, and one was caused by mixed organisms (Table 1). Thirteen patients had the results of bacterial culture tests at other hospitals. In six patients, the results of the bacterial culture test taken during our procedure were different from previous results. Therefore, changing antibiotics postoperatively was needed.

## 4. Discussion

Several studies have reported good results of treatment using an antibiotic-impregnated cement bead in chronic osteomyelitis [7,20]. In these studies, antibiotic-impregnated cement was mostly used as a temporary carrier of local antibiotics, followed by cement removal and definitive surgery.

Recent studies have reported the results of treatment using antibiotic-impregnated bone cement that fills dead spaces rather than beads. Ferrao et al. have reported the use of cement spacers for the definitive management of postoperative ankle infections [14]. In that study, the mid-term results of cement arthroplasty were good. Lee et al. have reported that primary cement arthroplasty might be a treatment option for ankle destruction in elderly and less active patients [15]. Kim et al. have reported a case of osteomyelitis after a calcaneal fracture treated with antibiotic-containing calcium phosphate cement [21]. No cement removal was performed, and the infection was controlled well.

In this study, antibiotic-impregnated cement filling was performed at the defected bone site after complete debridement of the infected tissue, rather than antibiotic-containing cement bead insertion. Antibiotic-containing cement beads have a wide contact surface to spread antibiotics but leave much dead space between the beads. Alternatively, antibiotic-impregnated cement filling leaves little dead space. Because antibiotic-containing cement is molded to fill the bone defect area, there is a supporting effect enough for patients to walk immediately after surgery. Moreover, no additional fixation is needed. Therefore, it helps in the rehabilitation of patients with prolonged chronic infection. Antibiotic bead insertion and the Masquelet technique require two-stage surgery, but the antibiotic-containing cement-filling technique can be completed in a single surgery [10].

For antibiotic-impregnated cement filling to be successful, complete debridement before the insertion of the cement is vital [15]. Performing bacterial culture correctly and administering the appropriate antibiotics postoperatively are also important. Antibiotic-impregnated cement insertion can reduce the duration of antibiotic use, which is generally recommended by infectious disease specialists. We stopped using antibiotics when the symptoms of infection were resolved and laboratory test results, such as ESR and CRP, improved. The suction drain inserted before wound closure allowed sufficient drainage.

Lang et al. (2021) reported that vancomycin-loaded calcium phosphate cement serves a dual role as a local antibiotic delivery system and a structural support in one-stage procedures. This aligns with our findings that antibiotic-mixed cement blocks can provide immediate weight-bearing capabilities and reduce the need for additional surgeries [22]. Additionally, studies from resource-limited settings, such as Gashegu et al. (2018) demonstrated that locally manufactured calcium sulfate cement impregnated with antibiotics achieved comparable outcomes to more advanced solutions, making it a cost-effective option. These findings support the adaptability and scalability of antibiotic-impregnated cement in diverse clinical environments [23].

In our hospital, until 2010, operative treatment was performed in two stages: antibiotic-impregnated cement bead filling, followed by cement removal and bone grafting. However, after 2010, antibiotic-impregnated cement filling was conducted as one definitive surgery. Infection was treated well in both groups. At the final follow-up, bone breakage or osteolysis around the cement was not observed after antibiotic-impregnated cement filling. Antibiotic-impregnated cement filling alone could control the infection and support structural stability. To minimize functional impairment, all patients were allowed to perform a range of motion exercises and full-weight-bearing walking, as tolerated, immediately after surgery without immobilization.

**Figure 1:**
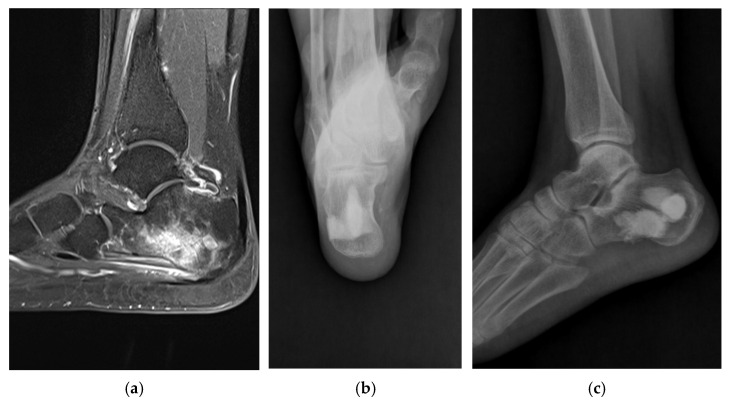
Antibiotic-mixed cement filling in a 57-year-old female patient (identified pathogen: Salmonella typhi). (**a**) Sagittal MRI shows chronic osteomyelitis on the calcaneus preoperatively. (**b**) Calcaneus axial radiograph shows that antibiotic-impregnated cement filling is well maintained 3 years postoperatively. (**c**) Calcaneus lateral radiograph shows that antibiotic-impregnated cement filling is well maintained 3 years postoperatively.

**Figure 2:**
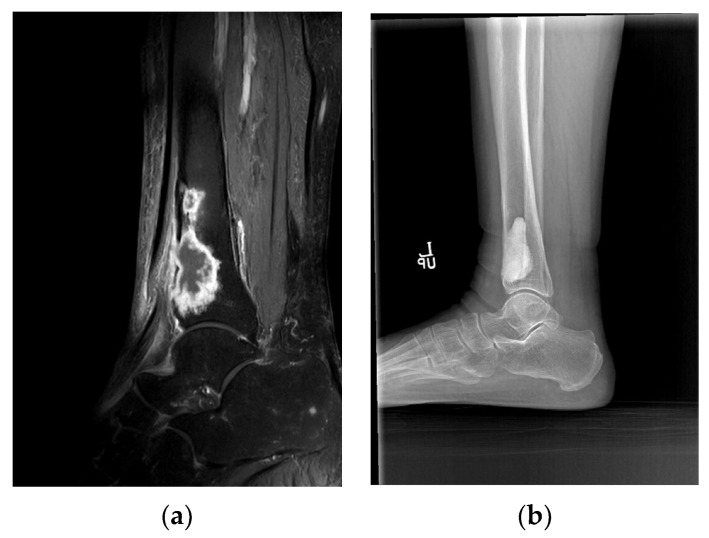
Antibiotic-mixed cement filling in a 75-year-old female patient (identified pathogen: Mycobacterium tuberculosis). (**a**) Sagittal MRI shows chronic osteomyelitis on the distal tibia shaft preoperatively. (**b**) Lateral radiograph shows that antibiotic-impregnated cement filling is well maintained 2 years postoperatively.

In three patients who had continued to receive antibiotic treatment at other hospitals, no bacteria were identified. Uncommon strains of microorganisms were identified, including Salmonella typhi (case 12) (Figure 1) and Mycobacterium tuberculosis complex (case 5) (Figure 2).

Most causes of infection were direct contamination, such as postoperative site (10 cases), open fracture (three cases), soft tissue laceration (one case), and puncture wound (two feet infection). Only two patients did not have any history of trauma or surgery: suspected hematogenous infection of Salmonella typhi (one patient), Mycobacterium tuberculosis complex (one patient).

**Figure 3:**
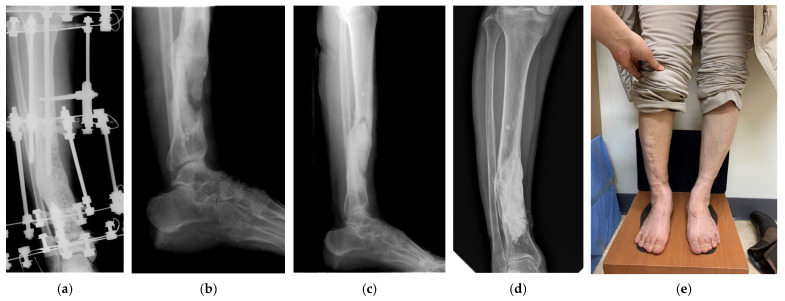
Antibiotic-mixed cement filling in a 41-year-old female patient (identified pathogen: Burkholderia cepacia, Bacteroides species, and Prevotella species). (**a**) Anteroposterior radiograph shows external fixation and antibiotic-impregnated cement bead insertion for chronic osteomyelitis of the distal tibia shaft in the previous hospital. (**b**) The lateral radiograph shows the removal of the external fixator and antibiotic-impregnated cement bead. (**c**) Lateral radiograph shows antibiotic-impregnated cement filling into the tibia shaft 2 weeks postoperatively. (**d**) Anteroposterior radiograph shows antibiotic-impregnated cement filling is well maintained 16 years postoperatively. (**e**) Medical photo shows bowing leg and no recurrence of infection 16 years postoperatively.

A female patient has been walking well even with a bowing leg for 16 years without recurrence of infection after cement-filling surgery for chronic osteomyelitis of the tibial shaft (case 6) (Figure 3). The patient underwent several surgeries for chronic osteomyelitis after surgery for fracture in another hospital. When the patient visited our hospital, an antibiotic-impregnated cement bead was inserted in the tibia, and an external fixator was applied (Figure 3a). After the removal of the external fixator and cement bead, debridement was performed in our hospital (Figure 3b). We irrigated the largely defected lesion continuously for 7 days (Figure 3c) and inserted an antibiotic-mixed cement block into the tibia (Figure 3d).

In a patient with cuboid infection involving the tarsometatarsal joint who underwent surgery seven times for osteomyelitis at other hospitals, even though the chronic infection was controlled successfully after our antibiotic-impregnated cement-filling surgery, serous discharge persisted for 30 days. In one patient with an infection of the first metatarsal head after hallux valgus surgery (case 17), we converted arthrodesis with autogenous bone graft after cement removal because serous discharge persisted for 74 days. However, in this patient, laboratory findings were normal, and no physical finding of infection was observed. After arthrodesis of the first metatarsophalangeal joint, the serous discharge stopped. It is thought that the shear force, not the axial loading force, between the cement and proximal phalangeal bone caused abnormal joint motion and serous discharge. Further study will be necessary on this hypothesis.

This study has some limitations. First, the antibiotics mixed with the cement were not specific to each microorganism. This is because the microorganism identification test was performed on the tissue collected during surgery. Further study on the effects of cement mixed with specific antibiotics is needed. Second, this study adopted a retrospective study design. The absence of a control group limits this study. Although two-stage procedures were performed in our hospital before 2010, data collection was inconsistent and not included. In the future, randomized controlled trials involving patients undergoing antibiotic-impregnated cement-filling surgery and traditional anti-bead temporary insertion for chronic osteomyelitis could help establish clinical practice guidelines. Lastly, the small number of cases (fewer than two per year) and the heterogeneity in patient characteristics (such as age, infection etiology, and surgical site) may have independently influenced clinical outcomes, thereby limiting the generalizability of our findings.

## 5. Conclusions

In conclusion, one-stage surgery with complete debridement and antibiotic-mixed cement filling is a relatively simple and effective treatment of intractable chronic osteomyelitis, which makes full-weight-bearing walking possible immediately after surgery.

## Figures and Tables

**Table 1:** Demographic characteristics of the 18 patients who underwent antibiotic-mixed cement filling for chronic osteomyelitis.

Case	Sex/Age	Site	Cause of Osteomyelitis	Infecting Organism from Intraoperative Debrided Tissue	Antibiotics (Duration, Days)		Discharge from Wound (Post-Op Days)	Normalization of CRP (Post-Op Days)	Normalization of ESR (Post-Op Days)
					Intravenous	Oral			
1	Female/54	Proximal tibia	Post-op (skin tumor excision)	No growth	Cefazolin (14)		Combined flap coverage	Normal, preoperative	Normal, preoperative
2	Male/32	Middle tibia	Open fracture	MRSA	Vancomycin (24)		14 (redo surgery)	18	25
3	Male/64	Distal tibia	Post-op (SMO)	MRSA	Vancomycin, Levofloxacin, Metronidazole (27)			20	30
4	Male/46	Distal tibia	Post-op (ORIF)	Streptococcus agalactiae	Cefazolin (7), Ampicillin (8)	Cephradine (14)		15	69
5	Female/75	Distal tibia	Unknown	Mycobacterium tuberculosis	Ceftezole (7)	HERZ (9 months)		24	190
6	Female/41	Distal tibia	Post-op (ORIF)	Burkholderia cepacia, Bacteroides and Prevotella	Piperacillin/Tazobactam (15), Ceftazidime (4)			32	Normal, preoperative
7	Male/39	Distal tibia	Open fracture	Pseudomonas aeruginosa	Ceftezole (10), Piperacillin/Tazobactam (18)	Cephradine (10)		27	49
8	Male/23	Calcaneus	Laceration	Escherichia vulneris	Cefazolin (26)			Normal, preoperative	Normal, preoperative
9	Male/53	Calcaneus	Open fracture	MRSA	Vancomycin, Rifampicin (15)	Ciprofloxacin (14)		6	22
10	Female/49	Calcaneus	Post-op (ORIF)	MSSA	Cefazolin, Rifampicin (8), Nafcillin (5)	Amoxicillin/Clavulanate, Ciprofloxacin (14)		Normal, preoperative	57
11	Male/57	Calcaneus	Post-op (ORIF)	MSSA	Ceftriaxone (6), Cefazolin (16)	Cephradine (10), Amoxicillin/Clavulanate, Ciprofloxacin (28)	28 (redo surgery)	14	14
12	Female/57	Calcaneus	Unknown	Salmonella typhi	Ceftriaxone (11)	Ciprofloxacin (14)		14	23
13	Male/43	Calcaneus	Post-op (ORIF)	MRSA	Ceftriaxone, Linezolid (8), Linezolid (4)	Linezolid (26)		38	56
14	Male/61	Calcaneus	Post-op (ORIF)	Pseudomonas aeruginosa	Ceftezole (5)	Cefditoren (7)		Normal, preoperative	30
15	Male/63	Cuboid	Puncture wound	Achromobacter xyloxidans	Ceftazidime (11)	Ciprofloxacin (14)	30 (healed with simple dressing)	3	27
16	Male/43	Medial cuneiform	Post-op (ORIF)	No growth	Amoxicillin/Sulbactam, Amikacin (29)	Amoxicillin/Sulbactam (7)		Normal, preoperative	12
17	Female/67	1st metatarsal bone	Post-op (HV)	MSSA	Nafcillin (21)	Cephradine (15)	74 (conversion into arthrodesis)	Normal, preoperative	45
18	Male/79	1st metatarsal bone	Puncture wound	No growth	Cefotiam (13)			Normal, preoperative	Normal, preoperative

Post-op, Postoperative; SMO, Supramalleolar osteotomy; ORIF, Open reduction and Internal fixation; HV, Hallux valgus; MRSA, Methicillin-resistant *Staphylococcus aureus*; MSSA, Methicillin-resistant *Staphylococcus aureus*; HERZ, Isoniazid, Ethambutol, Rifampin, Pyrazinamide.

## Data Availability

All data and code generated or analyzed during this study are included in this published article.
